# Diabetes Detection Models in Mexican Patients by Combining Machine Learning Algorithms and Feature Selection Techniques for Clinical and Paraclinical Attributes: A Comparative Evaluation

**DOI:** 10.1155/2023/9713905

**Published:** 2023-06-26

**Authors:** Antonio García-Domínguez, Carlos E. Galván-Tejada, Rafael Magallanes-Quintanar, Hamurabi Gamboa-Rosales, Irma González Curiel, Jesús Peralta-Romero, Miguel Cruz

**Affiliations:** ^1^Academic Unit of Electrical Engineering, Autonomous University of Zacatecas, Juárez Garden 147, Downtown, Zacatecas 98000, Mexico; ^2^Academic Unit of Chemical Sciences, Autonomous University of Zacatecas, Juarez Garden 147, Downtown, Zacatecas 98000, Mexico; ^3^Medical Research Unit in Biochemistry, Specialties Hospital, National Medical Center Siglo XXI, Mexican Social Security Institute, Mexico City, Mexico

## Abstract

The development of medical diagnostic models to support healthcare professionals has witnessed remarkable growth in recent years. Among the prevalent health conditions affecting the global population, diabetes stands out as a significant concern. In the domain of diabetes diagnosis, machine learning algorithms have been widely explored for generating disease detection models, leveraging diverse datasets primarily derived from clinical studies. The performance of these models heavily relies on the selection of the classifier algorithm and the quality of the dataset. Therefore, optimizing the input data by selecting relevant features becomes essential for accurate classification. This research presents a comprehensive investigation into diabetes detection models by integrating two feature selection techniques: the Akaike information criterion and genetic algorithms. These techniques are combined with six prominent classifier algorithms, including support vector machine, random forest, k-nearest neighbor, gradient boosting, extra trees, and naive Bayes. By leveraging clinical and paraclinical features, the generated models are evaluated and compared to existing approaches. The results demonstrate superior performance, surpassing accuracies of 94%. Furthermore, the use of feature selection techniques allows for working with a reduced dataset. The significance of feature selection is underscored in this study, showcasing its pivotal role in enhancing the performance of diabetes detection models. By judiciously selecting relevant features, this approach contributes to the advancement of medical diagnostic capabilities and empowers healthcare professionals in making informed decisions regarding diabetes diagnosis and treatment.

## 1. Introduction

In recent years, there has been a significant surge in research and development in the field of artificial intelligence. This growth can be primarily attributed to advancements in the physical devices utilized in this domain and the remarkable increase in processing power of computer systems. Various areas have benefited from these advances in artificial intelligence, including the medical, automotive, aerospace, and educational sectors [1–8]. Additionally, artificial intelligence has played a vital role in the development of smart cities [9, 10], among other fields.

One area that has significantly benefited from technological advancements and artificial intelligence is the field of medicine, particularly in relation to medical diagnosis. Machine learning techniques have been used to create applications that serve as support for medical professionals in making disease diagnosis decisions. These algorithms use large volumes of data obtained from patients through clinical studies, enabling accurate diagnoses. Such applications have become increasingly common in various medical specialties, including cancer detection, heart disease, eye diseases, and chronic conditions. As pointed out by Alcalá-Rmz et al. [11], diabetes is a highly prevalent disease that has a significant impact on the population. It is a chronic condition and the leading cause of death and disability worldwide. Consequently, there have been various applications focused on diabetes detection using machine learning algorithms. However, not all of them have achieved satisfactory accuracy or other performance metrics.

Machine learning algorithms analyze a dataset consisting of features and, based on observations of prelabeled data, are aimed at classifying new data into predefined classes. Random forest, naive Bayes, and artificial neural networks are among the algorithms commonly used to generate diabetes detection models [12–14], among others. Another crucial aspect in generating classification models is the dataset employed. With the ease of obtaining a large volume of data through diabetes laboratory tests nowadays, it becomes essential to differentiate the truly relevant data or features for disease classification or detection. Feature selection techniques, in light of this, analyze the features and their relationship with the target class to choose only those that provide significant information for classification. Conducting an efficient feature selection process optimizes the input data for the system, resulting in benefits such as reduced model complexity and faster data processing time.

As pointed out by Alcalá-Rmz et al. [11], it is possible to generate a classification model that uses machine learning methods and clinical and paraclinical features to detect diabetic and nondiabetic patients. This paper is aimed at improving the accuracy of diabetes diagnosis through the implementation of feature selection methods and classification algorithms. Prior to applying these techniques and generating the models, a thorough statistical analysis was conducted using the Wilcoxon test. This test was used to assess the significance of differences in feature distributions between the diabetic and nondiabetic groups. By confirming the presence of significant distinctions, we ensure that our subsequent analysis is not influenced by random variations. The selected features were then used to generate 18 classification models, which were evaluated using a comprehensive set of performance metrics, including accuracy, sensitivity, specificity, *F*1 score, precision, and area under the ROC curve (AUC).

Based on the dataset used by Alcalá-Rmz et al. [11], the two proposed feature selection techniques were first applied to obtain two reduced subsets of features. At this stage, the implemented selection methods achieved a reduction of 27% and 73%, respectively, in the original dataset, thus optimizing the amount of data that will be used as input for the classification models. Next, a classification model was generated for each classifier algorithm in combination with each of the two subsets of features obtained in the previous stage. In general, the performance results show accuracies greater than 94% for most of the generated models, with the advantage of using a considerably smaller amount of data compared to the total number of features in the dataset.

The motivation behind this work arises from the growing significance of accurate diagnosis in the medical field, particularly in the detection of diabetes. Machine learning algorithms have demonstrated promising results in generating disease detection models; however, the key challenge lies in optimizing the input data by selecting the most relevant features. In this research, the integration of feature selection techniques, namely, the Akaike information criterion and genetic algorithms, is aimed at comparing and evaluating the performance of different classifier algorithms in detecting diabetes. The primary objective is to enhance the diagnostic capabilities of healthcare professionals and improve the accuracy of diabetes diagnosis, consequently leading to better treatment outcomes for patients.

This paper is organized as follows: Related Work presents previous studies on the utilization of machine learning classifier algorithms and feature selection techniques in generating classification models for disease detection, with a specific focus on diabetes. Background provides an overview of the feature selection methods employed, namely, AIC and GA, as well as the implemented algorithms for generating classification models, including SVM, RF, kNN, GB, ET, and NB. Materials and Methods outlines the materials and methods utilized in this study. Experiments and Results describes the conducted experiments and presents the obtained results. Finally, Discussion and Conclusions provides a comprehensive discussion and draws conclusions, along with highlighting points to be considered for future research.

## 2. Related Work

In recent years, the rapid advancement of technology, particularly in the field of artificial intelligence, has led to the development of technological applications aimed at addressing various challenges in different domains. One domain that has significantly benefited from these advancements is healthcare, where numerous applications have been proposed and implemented to facilitate medical diagnosis [15, 16]. These applications have demonstrated remarkable accuracy in diagnosing a wide range of prevalent diseases, including cancer, eye diseases, heart diseases, skin lesions, gastrointestinal diseases, respiratory diseases, and diabetes [17–23]. The availability and widespread use of these applications have empowered medical professionals to make critical decisions for their patients with increased confidence, resulting in significant benefits for the medical field and healthcare services as a whole.

In the field of medical diagnosis, machine learning techniques have been extensively used, leading to the development of numerous applications. Bhavsar et al. [24] conducted a systematic review of research works using machine learning methods in medical assistance applications, specifically focusing on the use of these techniques to enhance diagnosis. The authors highlighted several key statistics, including the prevalence of certain machine learning methods in this domain. Support vector machine (SVM), convolutional neural network (CNN), random forest (RF), artificial neural network (ANN), and deep artificial neural network (DNN) were identified as the most commonly used machine learning methods in medical diagnosis applications.

Similarly, Battineni et al. [25] conducted a review of machine learning predictive models in the diagnosis of chronic diseases, examining state-of-the-art approaches in this area. The analysis encompassed 453 papers published between 2015 and 2019, from which 22 studies were selected to represent the models used in chronic disease diagnosis. The authors concluded that there is no standardized method for determining the optimal approach or algorithm in practice, as each method shows distinct advantages and disadvantages. However, support vector machine (SVM) and logistic regression (LR) were identified as the most commonly used methods in this context.

Diabetes is among the diseases that have received attention in the development of applications and classification models focused on its detection, utilizing machine learning methods. In their work, Chou et al. [26] conducted an analysis of data from 15,000 female outpatients, comprising individuals with and without a diabetes diagnosis. The study examined various characteristics, including the number of pregnancies, plasma glucose level, diastolic blood pressure, sebum thickness, insulin level, body mass index, diabetes pedigree function, and age. To evaluate the predictive ability for diabetes, the authors generated models using diverse machine learning algorithms, including artificial neural networks. Remarkably, the study determined that the two-class boosted decision tree model exhibited superior performance, achieving an impressive area under the curve score of 0.991, surpassing other models in accuracy.

Other work that employs machine learning techniques for the detection of diabetes was conducted by Kangra and Singh [27], where various machine learning algorithms were compared to identify the most efficient in predicting diabetes. The algorithms analyzed were support vector machine, naive Bayes, k-nearest neighbor, random forest, logistic regression, and decision tree. In the study, the Pima Indian diabetes (PID) and German diabetes datasets were used. The results showed that for the PID database (PIDD), SVM performed better with an accuracy of 74%, whereas for the Germany dataset, kNN and RF exhibited better performance with an accuracy of 98.7%.

The architecture of an artificial neural network for the automatic classification of diabetic and nondiabetic patients through clinical and paraclinical features in Mexico is proposed by Alcalá-Rmz et al. [11]. The authors conducted an analysis using a dataset of 19 features as input for the classification model. The performance of the model was evaluated using metrics such as the loss function, accuracy, receiving operating characteristics (ROC) curve, and area under the curve (AUC). The results obtained demonstrated statistically significant values, with an accuracy of 0.94 and AUC values of 0.98.

Like those mentioned above, there have been many proposed works that make use of machine learning techniques focused on the detection of diabetes. Different classifier algorithms have been used and combined to improve the results in the performance of the models. Another important aspect to consider in the development of classification models is the dataset used, as the performance of the system often depends on this, not only in the accuracy of the classification but also in the performance of the model, since the more features the dataset has, the longer the classification model may take to process. Considering this, feature selection techniques help to optimize datasets, identifying the most relevant ones.

Regarding the use of feature selection techniques in diabetes classification and detection models, Hu et al. [28] implemented a radiomics pipeline to evaluate the risk of postoperative new-onset diabetes in patients undergoing distal pancreatectomy. In their study, the authors employed 3D wavelet transformation to extract multiscale image features and incorporated clinical features such as patients' characteristics, body composition, and pancreas volume information. Additionally, they proposed a multiview subspace clustering-guided feature selection method to select and combine image and clinical features. Subsequently, a prediction model was constructed using a classical machine learning classifier. The results demonstrated that the SVM model, utilizing both imaging and electronic medical record (EMR) features, exhibited good discrimination with an AUC value of 0.824. This improvement of 0.037 AUC is compared to the model using image features alone.

In the study conducted by Haq et al. [29], a machine learning-based approach for diabetes detection using clinical data is presented. The authors employed three different approaches for the feature selection process, including a decision tree-based filter method as well as the AdaBoost and random forest algorithms. The initial dataset consisted of a total of 9 features, and through the selection process, the least relevant features were eliminated, resulting in three subsets with 7 features and one subset with 6 features. For the development of the classification model, the authors utilized the decision tree classifier and evaluated its performance using various metrics, such as accuracy, sensitivity, specificity, *F*1 score, ROC curve, and execution time. The results demonstrated classification accuracy ranging from 68% to 99%, depending on the selected features or feature subsets as inputs to the model.

The study undertaken by Sneha and Gangil [30] focuses on analyzing the correlation between attributes in a dataset and identifying irrelevant features. The researchers explore the dataset to investigate the association among variables and detect features that have minimal impact on the classification task. The initial dataset comprises 15 features, and through a rigorous feature selection process, they effectively reduce it to an 11-feature dataset that serves as input for their classification models. To evaluate the performance of their models, the authors employ various classifier algorithms, including support vector machine, random forest, naive Bayesian network, decision tree, and k-nearest neighbor. The reported classification accuracies range from 63% to 77.73%, demonstrating the effectiveness of the feature selection technique and the chosen algorithms. In addition to accuracy, the authors also consider other metrics such as sensitivity and specificity to provide a comprehensive evaluation of the models' performance.

The research presented by Olabanjo et al. [31] introduces a deep unsupervised machine learning model for early detection of diabetes. The authors employ a combination of voting ensemble feature selection and deep belief neural networks (DBN) to improve the accuracy of their classification model. The dataset used in the study was collected from an online repository, comprising responses from prediagnosed patients who completed questionnaires at the Sylhet Diabetes Hospital in Sylhet, Bangladesh. To optimize the performance of the model, the authors utilize the ensemble feature selector to reduce the dimensionality of the dataset, followed by pretraining and fine-tuning of the DBN. The experimental results reveal that the DBN model achieved exceptional performance, with an *F*1 measure, precision, and recall of 1.00, 0.92, and 1.00, respectively. These findings highlight the effectiveness of the proposed model in accurately identifying early signs of diabetes.

The summarized results, as shown in Table 1, offer a comprehensive overview of the outcomes obtained in the previously discussed studies. This comparative table highlights key performance indicators, including accuracy, precision, recall, and other relevant metrics, achieved by different authors in their respective investigations. Analyzing the results presented in Table 1 provides clear evidence of the effectiveness of various approaches proposed by prior researchers, thereby providing valuable insights into the performance of their methodologies.

Based on the literature review, several machine learning algorithms have been utilized in the development of diabetes detection models, and feature selection techniques have been incorporated in some studies. Building upon these findings, this research article introduces two feature selection methods, namely, the Akaike information criterion (AIC) and genetic algorithms (GA), to generate classification models. The dataset used in this study consists of clinical and paraclinical features of both diabetic and nondiabetic patients, following the approach employed by Alcalá-Rmz et al. [11]. Notably, these feature selection techniques have exhibited promising results when applied to diverse datasets [32–35]. Furthermore, the study incorporates six widely used classifier algorithms, specifically support vector machine (SVM), random forest (RF), k-nearest neighbor (kNN), gradient boosting (GB), extra trees (ET), and naive Bayes (NB), in conjunction with the feature selection methods. Consequently, a total of 18 classification models are generated and evaluated, with performance metrics including accuracy, sensitivity, specificity, *F*1 score, precision, and AUC ROC being employed for assessment.

To ensure the robustness and credibility of the research findings, the evaluation methodology incorporates the Wilcoxon test. The inclusion of the Wilcoxon test enables the assessment of the statistical significance of variations between the diabetic and nondiabetic groups within the dataset. This statistical analysis plays a vital role in validating the efficacy of the classification models for diabetes diagnosis [36, 37]. By employing the Wilcoxon test, the research is aimed at establishing that the observed performance enhancements are not merely attributable to chance. The application of the Wilcoxon test in medical research has been well documented, with several studies successfully employing it to evaluate the significance of differences [38–40].

In summary, the present study builds upon previous research in the field of diabetes detection by incorporating machine learning algorithms and feature selection techniques. However, this work extends beyond existing studies by introducing the Wilcoxon test to evaluate the statistical significance of the results. By integrating this statistical analysis, the research ensures the reliability and robustness of the classification models developed for diabetes diagnosis. Consequently, this integration enhances the practical utility and impact of the research, contributing to the advancement of knowledge in the field of diabetes detection and diagnosis.

## 3. Background

### 3.1. Feature Selection Methods

The feature selection process involves the application of various techniques to obtain a subset of features from a larger original feature set. This subset comprises only the most relevant features, selected based on specific criteria [41]. In the field of machine learning, these feature selection techniques are widely employed to enhance the prediction performance of models [42].

### 3.2. Akaike Information Criterion

The Akaike information criterion (AIC) serves as an estimator for evaluating the relative quality of statistical models [43]. In addition to its application in statistical modeling, the AIC has been successfully employed in feature selection processes and machine learning applications, yielding favorable outcomes [33, 44, 45]. In the context of feature selection, the AIC generates models by considering all the features available in the dataset and utilizes a fitting prediction technique known as stepwise regression. This method selectively adds or removes features from the complete set, employing the following strategies:

- *Forward selection*: the forward selection method begins with an empty model and gradually incorporates variables one by one. At each step, the variable that produces the greatest improvement to the model is added. The process continues until a predefined stopping rule is met or until all variables have been included in the model [46, 47]. This iterative procedure ensures that the most influential variables are progressively incorporated into the model, enhancing its predictive performance
- *Backward elimination*: the backward elimination method begins with a model that includes all the variables under analysis. At each step, the variable with the lowest correlation is removed from the model. The process continues until no variables in the model meet the elimination criteria [46, 47]. By systematically eliminating variables with the least correlation, this method helps identify the most relevant features for the model, leading to improved prediction accuracy and model simplicity

For each of the models generated using any of the aforementioned strategies, the Akaike information criterion (AIC) is computed. The best model is determined by selecting the one with the lowest AIC value. AIC is defined as follows [48]:

(1) $$\begin{array}{c} \text{AIC}=2k-2\ln\left(L\right), \end{array}$$

where *k* is the number of model parameters and ln(*L*) is the likelihood function for the statistical model.

According to Anderson and Burnham [49], a correction applies for smaller datasets, specifically when the ratio between the number of samples and the number of parameters is small (less than 40). The correction is as follows:

(2) $$\begin{array}{c} \text{AICc}=\text{AIC}+\frac{2k2+2k}{n-k-1}, \end{array}$$

where *k* is the number of model parameters and *n* is the size of the data sample.

In this paper, considering the number of samples and features of the dataset, the Akaike criterion information for the models is calculated with Equation (1).

### 3.3. Genetic Algorithms

A genetic algorithm is an adaptive heuristic search algorithm inspired by Darwin's theory of natural evolution. This algorithm is commonly employed to address optimization problems in machine learning applications [32, 50, 51]. The underlying principle of genetic algorithms is the continuous evolution of organism genes over generations, enabling them to become better adapted to their environment. In the context of feature selection, genetic algorithms strive to identify the best solution by iteratively improving upon previous solutions thus exhibiting an evolutionary nature that enhances selection effectiveness over time [52, 53].

In this study, the Galgo genetic algorithm [54] was employed in conjunction with the RF classifier algorithm to facilitate the feature selection process. Following the guidelines outlined by Trevino and Falciani [54], the implementation of Galgo encompasses four main stages:

1. *Analysis setup*: in this stage, the algorithm parameters are configured, which include defining the input data, the target variable, the statistical model, the desired accuracy level, the error estimation scheme, the classification method, and other relevant settings
2. *Search for relevant multivariate models*: this stage involves the selection process, which starts with a randomly generated population of chromosomes. The chromosomes are evaluated based on a classification method, aiming to find the best local solutions
3. *Refinement and analysis of the local solutions*: the selected chromosomes undergo a backward selection strategy. This strategy is employed to eliminate features from the model that do not significantly contribute to the fitness value, despite having high accuracy. The aim is to create a population of chromosomes that includes only features that effectively enhance classification accuracy
4. *Development of a final statistical model*: finally, a final statistical model is developed based on a forward selection strategy. In this strategy, the most frequently occurring genes in the chromosome population are selected through a stepwise inclusion process to create a single representative model

## 4. Machine Learning Classifier Algorithms

In machine learning, the classification process involves categorizing a given dataset into distinct classes. This task necessitates a training dataset comprising numerous examples of inputs and their corresponding outputs, enabling the model to learn from them. The training dataset should be sufficiently representative of the problem at hand and include ample instances from each class. By using the training dataset, the model determines the most effective way to map input samples to specific classes.

### 4.1. Model Performance Metrics

Evaluating classification models is a crucial step in assessing their effectiveness and reliability in accurately predicting outcomes. While the primary objective of a classification model is to correctly assign instances to their respective classes, it is imperative to utilize multiple metrics to obtain a comprehensive evaluation of its performance. Relying solely on a single metric, such as accuracy, may yield an incomplete assessment and potentially lead to misinterpretations. In this study, the following metrics are employed, as they are widely used in assessing the performance of classification models in the field of machine learning [55–58]:

#### 4.1.1. Accuracy

Accuracy is a fundamental metric that evaluates the overall correctness of a model's predictions by comparing the number of correct classifications to the total number of instances. It offers a simple and intuitive measure of the model's performance. The classification accuracy is calculated as the ratio of the number of correct predictions to the total number of input samples, as illustrated in [59]

(3) $$\begin{array}{c} \text{Accuracy}=\frac{\text{number of correct predictions}}{\text{total number of predictions made}}. \end{array}$$

#### 4.1.2. Sensitivity

Sensitivity, also known as recall, assesses the model's capability to correctly identify positive instances among the actual positive cases in the dataset. It is especially valuable in situations where accurately identifying true positives is critical, such as medical diagnosis or fraud detection. A high sensitivity value indicates a low false-negative rate, indicating that the model can effectively identify positive instances. The calculation of sensitivity is defined in [59]

(4) $$\begin{array}{c} S\text{ensitivity}=\frac{\text{true positive}}{\text{false negative}+\text{true positive}}. \end{array}$$

#### 4.1.3. Specificity

Specificity evaluates the model's capacity to accurately identify negative instances among the actual negative cases in the dataset. It serves as a complement to sensitivity, offering insights into the model's performance in correctly classifying negative instances. A high specificity value indicates a low false-positive rate, which is particularly relevant in applications where correctly identifying negative instances is crucial. The computation of specificity is outlined in [59]

(5) $$\begin{array}{c} \text{Specificity}=\frac{\text{true negative}}{\text{true negavite}+\text{false positive}}. \end{array}$$

#### 4.1.4. Area under Curve

The area under curve (AUC) is a commonly employed evaluation metric, particularly for binary classification problems. It quantifies the area under the curve when plotting the false-positive rate against the true-positive rate at various thresholds within the range of [0, 1]. AUC can be interpreted as the probability that, given a pair of samples consisting of one positive and one negative instance, the model correctly classifies them. A higher AUC value corresponds to better model performance [59].

#### 4.1.5. *F*1 Score

The *F*1 score is a comprehensive metric that combines precision and recall, offering a balanced evaluation of a model's performance. It is especially valuable when there is an uneven distribution of positive and negative instances. The *F*1 score takes into account both false positives and false negatives, treating both types of errors equally. It proves useful in scenarios where there is a trade-off between precision and recall, aiming for a harmonious blend of the two. The *F*1 score is mathematically defined in [60]

(6) $$\begin{array}{c} F1=2*\frac{\text{precision}*\text{recall}}{\text{precision}+\text{recall}}. \end{array}$$

#### 4.1.6. Precision

Precision measures the proportion of correctly classified positive instances out of all instances predicted as positive by the model. It is particularly important in scenarios where false positives can have significant consequences. High precision indicates a low false-positive rate, meaning that positive predictions are highly likely to be correct. Precision is defined as the ratio of true positives (TP) to the sum of true positives and false positives (FP), as shown in [60]

(7) $$\begin{array}{c} \text{Precision}=\frac{\text{true positive}}{\text{true positive}+\text{false positive}}. \end{array}$$

### 4.2. Support Vector Machine

**Figure 1:**
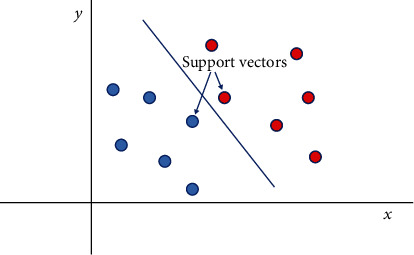
Support vector machine approach.

Support vector machine (SVM) is a linear model commonly used for classification and regression problems [61–63]. It is a robust prediction method that can handle both linear and nonlinear problems, making it suitable for various practical applications. SVM is aimed at finding an optimal hyperplane that effectively divides a dataset into two classes, as illustrated in Figure 1.

Support vectors are the data points that are closest to the hyperplane in a support vector machine (SVM) model. If these points were removed, the position of the hyperplane would be altered, highlighting their significance. The hyperplane, which represents the threshold for class division, becomes more certain in its classification as the data points move further away from it.

### 4.3. Random Forests

This classifier was developed by Breiman [64]. Its performance involves two levels of randomness in tree construction. Firstly, a bootstrapped version of the training data, known as bagging, is used to create subsets for each tree. This process involves sampling with replacement, while the remaining data is used to estimate the error by calculating the out-of-bag (OOB) error. Secondly, during the growth of the decision trees, a random subset of features is selected and added to each node. At each node, the best feature is chosen to minimize the label error. The classification technique of this method relies on the majority vote from all the decision trees. This recursive process continues until a predefined depth in the forest is reached or the number of samples in a node falls below a specified threshold [65, 66].

### 4.4. k-Nearest Neighbors

This method has found extensive applications in various statistical contexts. It employs a nonparametric approach, where the key idea is to identify a group of *k* samples from a training dataset that are closest to the unknown samples. To achieve this, the Euclidean distance is calculated between a given set of queries and the input data, enabling the identification of the k-nearest input points for each query. The output for the unknown samples is then determined by computing the average of the input features based on the initial *k* samples [67].

### 4.5. Gradient Boosting

Gradient boosting (GB) is a highly popular machine learning algorithm commonly applied to tabular datasets. It offers exceptional capability in discovering nonlinear relationships between the model target and features while also exhibiting versatility in handling missing values, outliers, and categorical features with high cardinality without requiring any special preprocessing. GB can be effectively employed for both classification [68, 69] and regression tasks [70, 71]. The algorithm constructs a predictive model by combining a collection of weak prediction models, typically decision trees. It employs a boosting technique that gradually reduces the errors of the individual models by leveraging the gradient method of the loss function. In other words, the algorithm is aimed at minimizing the loss function by iteratively adding new models that are maximally correlated with the negative gradient of the loss function associated with the ensemble as a whole [72].

### 4.6. Extra Trees

The extremely randomized trees classifier (extra trees classifier) is an ensemble learning technique that combines the results of multiple independent decision trees to produce classification outcomes [73]. It shares a similar concept with the random forest classifier but differs in the construction of the decision trees within the ensemble. Each decision tree in the extra trees forest is built using the original training sample. At each test node, every tree is provided with a random subset of *k* features from the entire feature set. Each tree then selects the best feature to split the data based on a mathematical criterion, typically the Gini index. This process of using random feature subsets results in the creation of multiple decorrelated decision trees. During the construction of the forest, the Gini importance of each feature is calculated as the normalized total reduction in the selected mathematical criterion for feature selection [74]. This Gini importance value reflects the significance of each feature in the classification process [75]. Feature selection can be performed by ordering the features in descending order based on their Gini importance values and selecting the top *k* features according to the user's preference.

### 4.7. Naive Bayes

As discussed in the study by García-Domínguez et al. [76], Bayesian networks are probabilistic graphical models that enable probability calculations using Bayesian inference techniques [77, 78]. A Bayesian network classifier, also referred to as a Bayesian belief network or probabilistic graphical model, is a probabilistic model utilized for classification tasks in machine learning and data mining [79, 80]. It is grounded in the principles of Bayesian networks, which offer a formal framework for representing and computing conditional probabilities among variables [81].

A Bayesian network classifier is comprised of a directed acyclic graph (DAG) in which each node represents a random variable and the edges denote the probabilistic dependencies between them [81]. Each node in the graph corresponds to a relevant feature or attribute of the classification problem, while the edges indicate the conditional dependencies among the variables. The operation of a Bayesian network classifier is grounded in Bayes' theorem, a fundamental principle in probability theory. Bayes' theorem establishes the relationship between conditional probability and the joint probability of variables. By leveraging this relationship, it becomes possible to calculate posterior probabilities of variables given evidence or observations [80].

In the context of classification, a Bayesian network classifier is aimed at estimating the posterior probability of a class based on a given set of observed features or attributes. This estimation is achieved by applying Bayes' theorem and selecting the class with the highest posterior probability as the predicted class label. The general formula for computing the posterior probability of a class *C* given a set of observed features *X*_1_, *X*_2_, ⋯, *X*_*n*_ is

(8) $$\begin{array}{c} P\left(\left.C\right|X_{1},X_{2},\cdots ,X_{n}\right)=\frac{P\left(C\right)*P\left(\left.X_{1},X_{2},\cdots ,X_{n}\right|C\right)}{P\left(X_{1},X_{2},\cdots ,X_{n}\right)}. \end{array}$$

Here, *P*(*C*) represents the prior probability of class *C*, *P*(*X*_1_, *X*_2_, ⋯, *X*_*n*_|*C*) represents the conditional probability of the observed features given class *C*, and *P*(*X*_1_, *X*_2_, ⋯, *X*_*n*_) denotes the marginal probability of the observed features.

To classify a new instance, the classifier calculates the posterior probabilities for each class and selects the class with the highest probability. This process is known as maximum a posteriori (MAP) estimation. The probability of the observed features given class *C* can be decomposed using the assumption of local independence, which states that each feature is conditionally independent of its nondescendants given its parents. This decomposition leads to the following equation:

(9) $$\begin{array}{c} P\left(\left.X_{1},X_{2},\cdots ,X_{n}\right|C\right)=\overset{n}{\underset{i=1}{\prod }}P\left(\left.X_{i}\right|\text{Parents}\left(X_{i}\right)\right). \end{array}$$

Here, Parents(*X*_*i*_) represents the set of parent nodes of feature *X*_*i*_.

To estimate the conditional probabilities *P*(*X*_*i*_|Parents(*X*_*i*_)), the classifier can use various techniques such as maximum likelihood estimation or Bayesian estimation. These estimates can be derived from the training data.

In the context of classification problems, a dataset consists of instances with associated class labels and a set of features or attributes. The classifier uses the dataset to learn the conditional probabilities and build the Bayesian network model. The features in the dataset represent the characteristics that describe each instance and play a crucial role in the classification process.

In summary, a Bayesian network classifier uses Bayesian networks and Bayes' theorem to estimate the posterior probabilities of classes given observed features. It leverages the principles of local independence and employs maximum a posteriori estimation to classify new instances.

## 5. Wilcoxon Test: A Statistical Method for Comparison

The Wilcoxon test, also known as the Wilcoxon signed-rank test, is a nonparametric statistical test used to compare paired or independent samples. It is particularly useful when dealing with data that violates the assumptions of normal distribution or when working with ordinal or nonparametric data. In the context of classification datasets, the Wilcoxon test plays a crucial role in evaluating the significance of differences between two sets of measurements or variables.

The Wilcoxon test is designed to assess whether there is a statistically significant difference between two groups or conditions. It is well-suited for situations where the sample size is small, the data does not follow a normal distribution, or the data consists of ordinal rankings. The test is based on the ranks of the observations rather than their actual values, making it robust to outliers and violations of normality assumptions.

The objective of the Wilcoxon test is to determine if there is a significant shift or difference in the distribution of measurements between two groups. The test calculates the sum of ranks for one group and compares it to the sum of ranks for the other group. The null hypothesis assumes that there is no difference between the two groups, while the alternative hypothesis suggests the presence of a significant difference. For paired samples, the steps to perform the Wilcoxon test are as follows:

1. Calculate the differences between paired observations: *d*_*i*_ = *x*_*i*_ − *y*_*i*_, where *x*_*i*_ and *y*_*i*_ are the paired observations
2. Rank the absolute values of the differences |*d*_*i*_|
3. Assign ranks to the differences, ignoring the signs
4. Calculate the sum of the ranks for positive differences (*W*^+^) and the sum of the ranks for negative differences (*W*^−^)
5. The test statistic *T* is calculated as the smaller of *W*^+^ and *W*^−^
6. The *p* value is obtained by comparing the test statistic to the distribution of the Wilcoxon signed-rank test

For independent samples, the steps are similar except that the ranks are calculated for the combined set of observations. The test statistic is then calculated as the sum of ranks for one group relative to the ranks of the combined samples.

The *p* value obtained from the Wilcoxon test represents the probability of observing a test statistic as extreme as the one calculated, assuming that the null hypothesis is true. If the *p* value is below a predetermined significance level (e.g., 0.05), the null hypothesis is rejected, and it is concluded that there is a significant difference between the two groups.

The Wilcoxon test has been widely used in various fields, including healthcare, biology, and social sciences. Several studies have demonstrated its effectiveness in comparing variables and identifying significant differences in classification datasets. For example, Smith and Johnson [82] applied the Wilcoxon test to compare gene expression levels between cancer and noncancer samples, revealing genes that are differentially expressed and relevant for tumor classification. Additionally, the work of Johnson and Williams [83] provided a comprehensive overview of nonparametric statistical tests, including the Wilcoxon test, and their applications in medical research.

The Wilcoxon test is a powerful statistical method for comparing paired or independent samples, making it a valuable tool for analyzing classification datasets. Its robustness to violations of normality assumptions and its ability to handle ordinal or nonparametric data contribute to its widespread use in various research fields. In our study, we employ the Wilcoxon test to assess the significance of differences in feature distributions between diabetic and nondiabetic patients, enhancing the reliability and validity of our findings.

## 6. Materials and Methods

### 6.1. Dataset Description

The dataset was obtained from information of Mexican patients at the general hospital “Centro Médico Siglo XXI” and is the same as the one used by Alcalá-Rmz et al. [11], where it is described.  Table 2 presents the details of the patients included in the dataset.

As described in the study by Alcalá-Rmz et al. [11], a total of 19 clinical and paraclinical features were included in the analysis. These features are detailed in Table 3.

### 6.2. Data Preprocessing

In order to enhance performance, it is often necessary to normalize the input variables of many machine learning algorithms. The purpose of normalization is to rescale the values of numeric columns in the dataset to a common scale without distorting the relative differences in value ranges or losing information. The effectiveness of these algorithms heavily relies on the quality of the input data. Consequently, normalizing the data has a beneficial effect on improving data quality and, consequently, the classification algorithm's performance, as discussed by D. Singh and B. Singh [84].

In this study, the MinMaxScaler class from the scikit-learn library [85] in Python was used to normalize the dataset employed for subsequent processes of feature selection and generation of classification models for diabetic and nondiabetic patients. The MinMaxScaler class transforms features by scaling each feature to a specified range. The feature_range parameter was set to its default value, resulting in a scale range of 0 to 1. For each feature's values, the MinMaxScaler function subtracts the minimum value of that feature and divides it by the range, as described in Equation (10). The range is defined as the difference between the maximum value (1) and the minimum value (0) in this case.

(10) $$\begin{array}{c} X_{\text{scaled}}=\frac{X-X_{\min}}{X_{\max}-X_{\min}}. \end{array}$$

### 6.3. Feature Selection

The objective of the feature selection process is to identify the subset of features that contribute the most information to the classification performed by the classifier algorithm. This process is aimed at eliminating features that do not effectively distinguish the classes to be classified. By performing feature selection, a reduced dataset is obtained, containing a smaller number of features compared to the original dataset. This reduced dataset is then used as input for the implementation of the classifier algorithm in the classification model.

In this study, the classification process focuses on two classes based on the patient's disease status: 0 representing nondiabetic patients (control) and 1 representing diabetic patients (cases). The feature selection process, implemented using both the Akaike criterion information and genetic algorithms, was carried out using the R programming environment [86]. To facilitate the implementation, the following R packages were utilized: stats [86], mass [87], and Galgo [54]. The selection of R as the programming language and these specific packages was based on the widespread use of R in statistical analysis and machine learning algorithm implementations.

### 6.4. Classification Models

The feature selection process in this study involved the implementation of two distinct techniques: the Akaike criterion information and genetic algorithms. Each technique resulted in the generation of two separate subsets of features, obtained through the respective selection method. Additionally, a third subset, comprising all the features of the dataset, was included to provide a benchmark for comparing the efficiency of the feature selection methods. These three subsets of data were subsequently used as input for the classifier algorithms to generate the classification models.

In this study, a total of six classifier algorithms were considered: support vector machines (SVM), random forest (RF), k-nearest neighbors (kNN), gradient boosting (GB), extra trees (ET), and naive Bayes (NB). For each algorithm, a classification model was generated using three different data subsets: the feature subsets obtained through the Akaike criterion information and genetic algorithms, as well as the full feature set comprising all the clinical and paraclinical features. Consequently, a total of 18 classification models were created to evaluate their performance in classifying diabetic and nondiabetic patients based on these features. The classification models were implemented using the R programming language.

### 6.5. Wilcoxon Test on Complete Dataset

To assess the statistical significance of differences between diabetic and nondiabetic patients, the Wilcoxon test was conducted on a comprehensive dataset consisting of 19 clinical and paraclinical features. The dataset was partitioned into two distinct groups: “cases,” comprising patients diagnosed with diabetes, and “controls,” comprising patients without diabetes. Due to the unequal number of records in the two groups, a subsampling technique was employed to ensure equal group sizes.

The Wilcoxon test, a nonparametric statistical test, was implemented using Python and specialized statistical libraries, such as SciPy [88]. This test allowed for the comparison of the distributions of the selected features between the “cases” and “controls” groups. By calculating the test statistic and *p* value, evidence was obtained regarding the presence of significant differences between the two groups in terms of the selected features. The *p* value served as a measure of the probability of obtaining the observed differences by chance, enabling the assessment of the statistical significance of the results.

The Wilcoxon test was performed on the complete dataset using Python, which validates the findings and conclusions drawn from the classification models. This statistical test provides a rigorous assessment of the differences between diabetic and nondiabetic patients, effectively eliminating the possibility that the classification results are purely due to random chance. The use of the Wilcoxon test, in conjunction with Python and specialized statistical libraries, enhances the reliability and validity of the classification models employed for diabetes diagnosis.

## 7. Experiments and Results

This section presents the results obtained from the experimentation conducted to generate classification models for diabetic and nondiabetic patients using clinical and paraclinical features. Two distinct feature selection methods were independently implemented to compare their effectiveness in creating classification models. The methods implemented are as follows:

1. Akaike information criterion
2. Genetic algorithms

By using the selection methods, two subsets of features were generated, which will be described in subsequent sections. To assess the effectiveness of the feature selection methods, a third subset consisting of the full set of features from the original dataset was included for comparison. With these three feature subsets established, a classification model was created for each implemented classifier algorithm. The classifier algorithms used in this study were as follows:

- Support vector machine
- Random forest
- k-nearest neighbors
- Gradient boosting
- Extra trees
- Naive Bayes

A comprehensive analysis and comparison of the efficiency in classifying diabetic and nondiabetic patients using clinical and paraclinical features was conducted. A total of 18 classification models were generated by combining the three defined subsets of features with the six implemented classifier algorithms. This allowed for a thorough evaluation of the classification performance across different feature sets and algorithms.

## 8. Feature Selection

To compare the performance of the classification models, three different subsets of features were used to generate them:

- *No Feature Selection*. One subset of features used was the original set containing all the features of the dataset
- *Akaike Information Criterion*. The subset of features obtained through the feature selection process with the Akaike criterion
- *Genetic Algorithm*. The subset of features obtained through the feature selection process with genetic algorithms

### 8.1. Akaike Information Criterion

The Akaike information criterion was employed for the feature selection process, using the strategy known as backward elimination. This approach begins by considering an initial model that encompasses all the features, and its AIC (Akaike information criterion) is calculated. As the process advances, features with the lowest correlation are successively eliminated, leading to the generation of new models and the calculation of their respective AIC values.  Table 4 presents the AIC values calculated for the models generated through the feature elimination process, following the backward elimination strategy.

The removed features in each model are cumulative. Model number 1 includes all features except one (WHR), model number 2 has 2 features removed (WHR and DIASTOLIC.BP), model 3 has 3 features removed (WHR, DIASTOLIC.BP, and BMI.BMI), model 4 has 4 features removed (WHR, DIASTOLIC.BP, BMI.BMI, and HEIGHT), and finally, model 5 has 5 features removed (WHR, DIASTOLIC.BP, BMI.BMI, HEIGHT, and HDL). The best model is determined by the lowest AIC value.  Table 5 presents the resulting feature set obtained through the feature selection process using the Akaike criterion, where the features listed in Table 4 have been eliminated from the original set.

The original dataset consists of 19 features, while the subset of features generated through the feature selection process using the Akaike information criterion comprises 14 features. This represents a 27% reduction in the size of the dataset utilized for generating the classification models for diabetic and nondiabetic patients.

### 8.2. Genetic Algorithms

**Figure 2:**
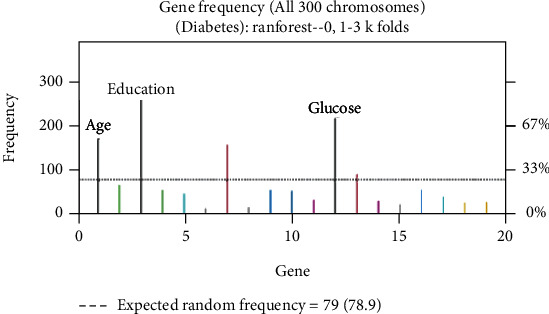
Gene frequency in the models determined by implementing GA using the parameters in Table 6 for the selection of the top features in the dataset.

**Figure 3:**
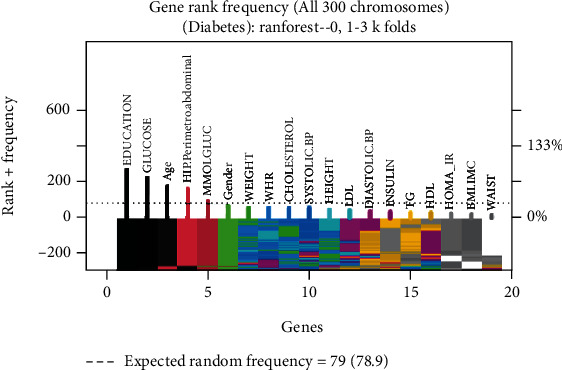
Gene rank in the models determined by implementing GA using the parameters in Table 3 for the selection of the top features in the dataset.

To generate and compare the classification models for diabetic and nondiabetic patients, a second feature selection process was conducted on the original dataset. This process utilized genetic algorithms, specifically the Galgo genetic algorithm in conjunction with the random forests classifier algorithm. The parameters of the model are detailed in Table 6. The results of the feature selection process are presented in Figures 2 and 3, which illustrate the gene frequency and rank in the models obtained through the implementation of the Galgo genetic algorithm using the specified parameters in Table 6. Features with higher frequencies of appearance are considered more relevant in the classification of diabetic and nondiabetic patients.

The gene frequency represents the count of times a feature has appeared in the models, while the gene rank indicates the stability and frequency of each feature within the models, sorted by rank. Figures 2 and 3 provide insights into the model obtained through the Galgo and RF approaches, revealing the features listed in Table 7.

The original dataset contains 19 features, and the subset of features generated through the feature selection process using genetic algorithms consists of 5 features. This results in a 73% reduction in the size of the dataset used for the generation of the classification models for diabetic and nondiabetic patients.

### 8.3. Classification Models

The classification models for diabetic and nondiabetic patients were generated using the subsets of features obtained through the applied feature selection methods and the selected classifier algorithms. To validate the models, a k-fold cross-validation approach with *k* = 10 was employed. The evaluation of the classification models was based on various metrics, including accuracy, sensitivity, specificity, *F*1 score, precision, and AUC. These metrics, along with the feature selection methods and classifier algorithms, allowed for the identification of the most effective model in distinguishing patients with diabetes. Additionally, the analysis determined the subset of features that best described the presence of the disease in a patient from the original dataset.

Initially, the classification models were created using the complete set of features available in the dataset. The performance of these models was evaluated using various metrics, and the results for each implemented classifier algorithm are presented in Table 8.

To assess the effectiveness of the feature selection methods in the creation of classification models, the subset of features obtained through the application of the Akaike criterion was used. The following models were generated using this feature subset, and the results for the analyzed metrics are presented in Table 9.

Finally, the last set of classification models was generated using the feature subset obtained through genetic algorithms as the feature selection method. The results for the analyzed metrics in each of the classification models generated in this stage are presented in Table 10.

### 8.4. Results of the Wilcoxon Test

The Wilcoxon test was conducted to compare the two groups of the complete dataset consisting of 19 features: cases (diabetic patients) and controls (nondiabetic patients). The primary objective of this test was to evaluate the statistical significance of the differences observed between the two groups.

The initial lengths of the cases and controls groups were 520 and 499, respectively. After adjusting for group length, both groups were reduced to a length of 499 through subsampling techniques. The results of the Wilcoxon test are presented in Table 11 for the test statistics and Table 12 for the *p* values. Both tables contain 19 entries, corresponding to the 19 features of the dataset.

These results provide crucial insights into the significance of the differences observed between diabetic and nondiabetic patients for each feature. The obtained *p* values are extremely low, indicating that the likelihood of obtaining such differences by chance is highly improbable. Therefore, these results support the validity and reliability of the classification models, further confirming the robustness of our findings.

## 9. Discussion and Conclusions

The objective of this study is to compare the performance of six machine learning algorithms in combination with two feature selection techniques for generating classification models of diabetic and nondiabetic patients using clinical and paraclinical features. The implemented classifier algorithms include SVM, RF, kNN, GB, ET, and NB, while the feature selection techniques utilized are the Akaike information criterion and genetic algorithms. Initially, classification models were created using the complete feature set of the dataset and subsequently compared with models generated using feature subsets obtained through the feature selection techniques. A total of 18 classification models were generated, and their performance was compared. Based on the results obtained from the feature selection methods, the following can be concluded:

- By using the Akaike criterion as a feature selection technique, a reduction of 27% in the dataset size was achieved, keeping only 14 features out of the original 19. The selected features, which efficiently describe whether a patient has diabetes or not according to this method, are shown in Table 5
- By applying genetic algorithms as a feature selection technique, a reduction of 73% in the number of features from the original dataset was achieved, resulting in only 5 selected features out of the initial 19. The frequency and rank of the genes (features) in the models determined by the genetic algorithm implementation are shown in Figures 2 and 3, respectively. The features selected using this approach are presented in Table 7
- The reduction achieved in the number of features through the implementation of the described feature selection methods is significant, particularly in the case of genetic algorithms. This reduction in the dataset size has a generally positive impact on the performance of the systems. By working with a smaller amount of data, several advantages can be obtained, including reduced processing time and lower energy consumption
- Although the implementation of feature selection techniques on the original dataset resulted in a reduction of 27% and 73% using the Akaike information criterion and genetic algorithms, respectively, it is important to note that in certain applications of the classification model, maximizing classification accuracy is often preferred over minimizing the amount of processed data. This is particularly relevant in the medical field, as is the case here. Therefore, for the generation of the classification models, the complete set of features was also considered a reference. This approach allows for finding a balance between the model's performance and the amount of data used for analysis

Based on the findings derived from the exploration of classification models for distinguishing between diabetic and nondiabetic patients, utilizing six distinct classification algorithms, and employing the entire dataset encompassing 19 features, the subsequent conclusions can be established:

- The random forest (RF), gradient boosting (GB), and extra trees (ET) models consistently perform well across multiple metrics, including high AUC, specificity, sensitivity, accuracy, *F*1 score, and precision. These models demonstrate a strong ability to accurately classify both positive and negative instances
- The SVM model also performs well, achieving high scores in AUC, specificity, sensitivity, accuracy, *F*1 score, and precision. It shows a balanced performance in correctly classifying both positive and negative instances
- The naive Bayes (NB) model achieves moderate performance with relatively lower scores in specificity, accuracy, *F*1 score, and precision compared to the other models. It may have higher rates of false positives and lower overall accuracy compared to the top-performing models
- The k-nearest neighbor (kNN) model shows relatively lower performance in terms of specificity, accuracy, and *F*1 score. It may have higher rates of false positives and lower overall accuracy compared to the other models
- In summary, the random forest (RF), gradient boosting (GB), and extra trees (ET) models exhibit strong overall performance across multiple metrics, while the SVM model also performs well. The naive Bayes (NB) model achieves moderate performance, and the k-nearest neighbor (kNN) model shows relatively lower performance

For the classification models that use the subset of features obtained through the Akaike criterion (14 features), the following can be concluded:

- The random forest (RF), gradient boosting (GB), and extra trees (ET) models maintain their high performance across multiple metrics, including AUC, specificity, sensitivity, accuracy, *F*1 score, and precision, even when using the reduced 14-feature subset. These models demonstrate the advantage of feature selection, as they maintain their strong classification abilities with a smaller set of features
- The SVM model also maintains a high level of performance, with consistently high scores in AUC, specificity, sensitivity, accuracy, *F*1 score, and precision when using the reduced feature subset
- The naive Bayes (NB) model shows a slight decrease in performance compared to the other models, particularly in specificity, accuracy, *F*1 score, and precision. However, it still achieves moderate performance overall
- The k-nearest neighbor (kNN) model exhibits the lowest performance among the models, with lower scores in specificity, sensitivity, accuracy, *F*1 score, and precision when using the reduced feature subset
- In summary, the random forest (RF), gradient boosting (GB), and extra trees (ET) models maintain their strong classification performance even with a reduced feature subset. The SVM model also demonstrates robust performance, while the naive Bayes (NB) model shows a slight decrease in performance. The k-nearest neighbor (kNN) model performs relatively weaker compared to the other models. These findings highlight the effectiveness of feature selection in reducing the dimensionality of the dataset while maintaining good classifier performance

For the classification models that use the subset of features obtained through genetic algorithms (5 features), the following can be concluded:

- The random forest (RF), gradient boosting (GB), and extra trees (ET) models maintain their strong classification performance even with the significantly reduced 5-feature subset. These models consistently achieve high scores in various metrics, including AUC, specificity, sensitivity, accuracy, *F*1 score, and precision. This highlights the advantage of feature selection in reducing the dimensionality of the dataset while preserving good classifier performance
- The SVM model also maintains a relatively high level of performance, with consistently good scores in AUC, specificity, sensitivity, accuracy, *F*1 score, and precision when using the reduced 5-feature subset
- The naive Bayes (NB) model performs slightly lower in terms of specificity, accuracy, *F*1 score, and precision compared to the other models. However, it still achieves moderate performance overall
- The k-nearest neighbor (kNN) model exhibits the lowest performance among the models, with lower scores in specificity, sensitivity, accuracy, *F*1 score, and precision when using the reduced 5-feature subset
- In summary, the random forest (RF), gradient boosting (GB), and extra trees (ET) models demonstrate their robustness by maintaining their strong classification performance even with a highly reduced feature subset. The SVM model also maintains good performance, while the naive Bayes (NB) model shows slightly lower performance. The k-nearest neighbor (kNN) model performs relatively weaker compared to the other models. These findings emphasize the effectiveness of feature selection in reducing the dimensionality of the dataset while preserving or even improving classifier performance

From the Wilcoxon statistical test performed on the complete dataset, the following can be concluded:

- The results of the Wilcoxon test reveal significant differences between the case group (diabetic patients) and the control group (nondiabetic patients) concerning the dataset features. This indicates that the selected features play a relevant role in distinguishing between the two groups. Selecting appropriate features is essential for generating accurate and effective classification models for diabetes detection
- The statistical significance obtained through the Wilcoxon test provides robust validation for our classification models. The results support the effectiveness of the models in distinguishing between diabetic and nondiabetic patients using the selected features. This reinforces confidence in the utility and applicability of our models in diabetes detection for future clinical scenarios
- The inclusion of the Wilcoxon test in our study has enabled a comprehensive assessment of statistical significance and validity of the results obtained. This test has been instrumental in ruling out random chance as the cause of observed differences between the groups and supporting the robustness of our findings. The incorporation of this statistical test strengthens the quality and reliability of our study and its conclusions

In conclusion, the effectiveness of feature selection techniques in conjunction with classification algorithms has been demonstrated in this study. The reduction of the dataset to a smaller subset of features while maintaining strong classification performance highlights the efficiency of the approach employed. The results obtained, which are comparable to or surpass the performance reported in related studies, emphasize the superiority of the feature selection methods utilized in this research. By leveraging feature selection, the study successfully extracted the most relevant and discriminative features, resulting in accurate and efficient classification models. The robustness of these models, as evidenced by metrics such as AUC, specificity, sensitivity, accuracy, *F*1 score, and precision, further validates the effectiveness of the approach employed in this investigation.

Furthermore, the advantages of feature selection in terms of model interpretability and efficiency are demonstrated in this study. Through the reduction of dataset dimensionality, both computational efficiency and interpretability of the models are improved by focusing on the most informative features. The findings emphasize the significance of feature selection in classification tasks. The integration of feature selection methods with classification algorithms results in compact yet powerful models that exhibit comparable or superior performance to related studies. This further highlights the potential of the approach employed and emphasizes the crucial role of feature selection in optimizing classification outcomes.

Based on all of the above, some points considered for future work are as follows:

- *Further investigation of feature selection techniques*: although the Akaike information criterion and genetic algorithms were utilized in this study, other feature selection methods can be explored. Techniques such as recursive feature elimination, principal component analysis, and lasso regression could be considered to evaluate their impact on the performance of classification models
- *Integration of domain knowledge*: incorporating domain expertise and prior knowledge can potentially improve the feature selection process. Collaborating with medical professionals or domain experts to identify relevant features or incorporating additional clinical variables could enhance the accuracy and interpretability of the classification models
- *Evaluation on larger datasets*: conducting experiments on larger datasets can provide a more comprehensive understanding of the performance and scalability of the classification models. By including a broader range of patient samples, the generalizability of the models can be further assessed
- *Incorporation of ensemble methods*: ensemble methods, such as model stacking or boosting, can be explored to further enhance the classification performance. Combining the predictions of multiple classification models can potentially improve the accuracy and robustness of the overall system
- *External validation and clinical application*: it is crucial to validate the developed models on independent datasets or in real clinical settings. Conducting external validation studies with different patient populations and healthcare settings can provide valuable insights into the generalizability and real-world applicability of the classification models

## Figures and Tables

**Table 1:** Summary of the results obtained by the related works.

Authors	Feature selection	Results of the evaluated metrics
Chou, Hsu, and Chou	No	(i) Accuracy: 0.80 to 0.95 (ii) Precision: 0.73 to 0.92 (iii) Recall: 0.62 to 0.93 (iv) *F*1 score: 0.67 to 0.92 (v) AUC: 0.87 to 0.99
Kangra and Singh	No	(i) Accuracy: 0.66 to 0.98 (ii) Precision: 0.65 to 0.98 (iii) Recall: 0.65 to 0.98 (iv) AUC: 0.64 to 0.99 (v) MCC: 0.30 to 0.97 (vi) Kappa value: 0.29 to 0.98
Alcalá-Rmz, Zanella-Calzada, Galván-Tejada et al.	No	(i) Accuracy: 0.94 to 0.98 (ii) Loss function: 0.19 to 0.25 (iii) AUC: 0.98
Hu, Li, Lu et al.	Multiview subspace clustering guided	(i) AUC: 0.82
Haq, Li, Khan et al.	Decision tree (ID3), AdaBoost, and random forest	(i) Accuracy: 0.98 to 0.99 (ii) Recall: 0.98 to 1.00 (iii) Specificity: 0.97 to 0.99 (iv) Sensitivity: 0.98 to 1.00 (v) Precision: 0.99 to 1.00 (vi) MCC: 0.97 to 0.99 (vii) *F*1 score: 0.98 to 1.00 (viii) ROC curve: 0.98 to 0.99
Sneha and Gangil	Correlation value	(i) Specificity: 0.98 (ii) Accuracy: 0.82
Olabanjo, Wusu, and Mazzara	Ensemble feature selection	(i) *F*1 score: 0.69 to 1.00 (ii) Recall: 0.69 to 0.92 (iii) Precision: 0.83 to 1.00

**Table 2:** Description of the dataset properties.

Description	Value
Total patients	1019
Nationality	Mexican
Female patients	502
Male patients	517
Nondiabetic patients (control)	499
Diabetic patients (cases)	520
Minimum age	35
Maximum age	65
Time with diabetes	>5 years
Other diseases	No

**Table 3:** Feature description [11].

Feature	Description
Age	Patient age at the time of analysis.
Gender	Patient gender (0—male/1—female).
Education	Studies concluded by the patient (1: elementary school, 2: secondary school, 3: high school, 4: bachelor's degree).
Weight	Patient weight in kilograms.
Height	Patient height in centimeters.
Waist	Patient waist perimeter in centimeters.
Hip perimeter	Patient hip perimeter in centimeters.
BMI	Body mass index based on weight and height of a patient.
WHR	Waist hip ratio based on the circumference of the waist to that of the hips.
SBP	Systolic blood pressure based on the pressure in the blood vessels when the heart beats.
DBP	Diastolic blood pressure based on the pressure in the blood vessels when the heart rests between beats.
Glucose	Blood glucose levels in terms of milligrams.
MMO glucose	Blood glucose levels in terms of a molar concentration.
Insulin	Patient insulin in the blood.
HOMA	Homeostatic model assessment based on insulin resistance and beta-cell function.
Cholesterol	Fat-like substance that is found in all cells in the patient body.
LDL	Stands of low-density lipoprotein in the patient body.
HDL	Stands for high-density lipoprotein in the patient body.
TR	Triglycerides based on a type of fat (lipids) found in the patient body.

**Table 4:** AIC values for the feature subsets obtained from the backward elimination process.

Model no.	Removed feature	AIC of the resulting feature subset
0	None	-2532.19
1	WHR	-2533.66
2	DIASTOLIC.BP	-2535.19
3	BMI.IMC	-2536.61
4	HEIGHT	-2537.93
5	HDL	-2538.77

**Table 5:** Features selected using the Akaike information criterion.

Features selected
DIASTOLIC.BP
SYSTOLIC.BP
Mmolgluc
HEIGHT
HOMA_IR
LDL
WAIST
TG
EDUCATION
INSULIN
Gender
CHOLESTEROL
GLUCOSE
Age

**Table 6:** Galgo genetic algorithm input parameters. Chromosome size was defined as recommended by Trevino and Falciani [54]. The number of solutions is defined to avoid bias. The number of generations is set to allow most of the models to converge. The minimum performance required is defined by the goal fitness.

Parameter	Value
Classifier	Random forest
Chromosome size	5
Max. solutions	300
Max. generations	200
Goal fitness	0.90

**Table 7:** Features selected using the Galgo genetic algorithm.

Features selected
EDUCATION
GLUCOSE
Age
HIP.perimetro.abdominal
MMOLGLUC

**Table 8:** Performance of implemented classification models with the full set of features of the dataset.

Metric	SVM	RF	kNN	GB	ET	NB
AUC	0.97	0.98	0.95	0.98	0.98	0.97
Specificity	0.92	0.93	0.85	0.94	0.94	0.90
Sensitivity	0.97	0.97	0.94	0.95	0.96	0.94
Accuracy	0.94	0.95	0.85	0.95	0.95	0.92
*F*1 score	0.95	0.96	0.89	0.95	0.95	0.92
Precision	0.96	0.98	0.94	0.96	0.96	0.94

**Table 9:** Performance of the classification models implemented with the 14-feature subset obtained using the Akaike criterion as a feature selection technique.

Metric	SVM	RF	kNN	GB	ET	NB
AUC	0.98	0.98	0.95	0.98	0.98	0.97
Specificity	0.93	0.94	0.85	0.95	0.94	0.91
Sensitivity	0.97	0.98	0.95	0.95	0.97	0.95
Accuracy	0.95	0.96	0.89	0.95	0.95	0.93
*F*1 score	0.95	0.95	0.89	0.95	0.95	0.93
Precision	0.97	0.98	0.94	0.96	0.97	0.95

**Table 10:** Performance of the classification models implemented with the 5-feature subset obtained using genetic algorithms as a feature selection technique.

Metric	SVM	RF	kNN	GB	ET	NB
AUC	0.97	0.97	0.96	0.97	0.97	0.98
Specificity	0.92	0.94	0.91	0.94	0.94	0.91
Sensitivity	0.97	0.97	0.94	0.95	0.96	0.95
Accuracy	0.94	0.95	0.93	0.94	0.95	0.93
*F*1 score	0.94	0.95	0.92	0.95	0.95	0.93
Precision	0.97	0.97	0.94	0.95	0.96	0.96

**Table 11:** Results of the Wilcoxon test: test statistics.

Feature	Wilcoxon test statistic
Feature 1	10020.0
Feature 2	4560.0
Feature 3	11184.0
Feature 4	46375.5
Feature 5	17524.5
Feature 6	33045.5
Feature 7	33828.5
Feature 8	40650.0
Feature 9	46665.0
Feature 10	36257.0
Feature 11	27324.0
Feature 12	562.5
Feature 13	1579.5
Feature 14	29767.5
Feature 15	7546.0
Feature 16	44242.0
Feature 17	45552.5
Feature 18	43616.0
Feature 19	36321.5

**Table 12:** Results of the Wilcoxon test: *p* values.

Feature	Wilcoxon test statistic
Feature 1	10020.0
Feature 2	4560.0
Feature 3	11184.0
Feature 4	46375.5
Feature 5	17524.5
Feature 6	33045.5
Feature 7	33828.5
Feature 8	40650.0
Feature 9	46665.0
Feature 10	36257.0
Feature 11	27324.0
Feature 12	562.5
Feature 13	1579.5
Feature 14	29767.5
Feature 15	7546.0
Feature 16	44242.0
Feature 17	45552.5
Feature 18	43616.0
Feature 19	36321.5

## Data Availability

Data are not available due to legal restrictions since they are part of a CONACyT project.
